# Inactivating *BTK* mutations in large B‐cell lymphoma in a real‐world cohort: Strong correlation with *BCL2* translocation

**DOI:** 10.1002/jha2.489

**Published:** 2022-06-24

**Authors:** Lone Schejbel, Marie Fredslund Breinholt, Anne Ortved Gang, Torsten Holm Nielsen, Lars Møller Pedersen, Estrid Høgdall, Peter Nørgaard

**Affiliations:** ^1^ Department of Pathology Copenhagen University Hospital Herlev Denmark; ^2^ Department of Pathology Copenhagen University Hospital Rigshospitalet Denmark; ^3^ Department of Haematology Copenhagen University Hospital Rigshospitalet Denmark; ^4^ Department of Haematology Zealand University Hospital Roskilde Denmark

**Keywords:** BTK, molecular pathology, non‐Hodgkin lymphoma

## Abstract

Inactivating mutations in Bruton's tyrosine kinase (*BTK*) in patients with follicular lymphoma (FL) have recently been reported. These mutations were found in BTK inhibitor‐treatment naïve patients. Here, we report the *BTK* mutation status in a real‐world cohort of patients with non‐Hodgkin lymphoma. We found primary *BTK* mutations in 7.7% of patients with large B‐cell lymphoma (LBCL) and in 14.1% of patients with FL. All patients with *BTK*‐mutated LBCL were *BCL2* translocation positive, and the correlation between *BCL2* translocation and *BTK* mutation persisted even when patients with known transformation from FL were excluded.

## 1 INTRODUCTION

Constitutive activation of the B‐cell receptor (BCR) and signaling through Bruton's tyrosine kinase (BTK) has traditionally been considered mandatory in B‐cell lymphomas. However, in diffuse large B‐cell lymphoma (DLBCL), cell lines of the activated B‐cell‐like (ABC) subtype have been shown to be dependent on BTK signaling, while cell lines of the germinal center B‐cell‐like (GCB) subtype were independent of BTK [1]. The introduction of BTK inhibitors as part of the treatment regimen in chronic lymphatic leukemia (CLL), lymphoplasmacytic lymphoma (LPL) and mantle cell lymphoma (MCL) has been very successful [2]. BTK inhibitor therapy has also been tested in DLBCL patients in combination with rituximab, cyclophosphamide, doxorubicin, vincristine, and prednisone (R‐CHOP) therapy in several clinical trials with varying success [3, 4, 5, 6]. The effect may depend on the subtype and age of the patients, as the BTK inhibitor ibrutinib plus R‐CHOP seems to improve event‐free survival and overall survival in younger patients (<60 years of age) with the ABC subtype [7].

In patients with X‐linked hypogammaglobulinemia (XLA), inactivating germline mutations in *BTK* result in an immunodeficiency characterized by hypogammaglobulinemia and a decreased number of B lymphocytes, which causes early onset of severe bacterial infections in the patients. The mutations causing XLA are scattered throughout the coding regions, with many unique mutations and some recurrent hot spots [8].

## 2 PATIENTS AND METHODS

The study included an unselected cohort of 320 non‐Hodgkin lymphoma patients subjected to next‐generation sequencing (NGS) as part of the routine diagnostic workflow at Herlev Hospital during a 2‐year period from 1 January 2020 to 1 January 2022. NGS analysis was performed with a custom‐designed targeted lymphoma panel using Ampliseq Ion Torrent technology (Thermo Fisher Scientific). Our NGS assay included all coding exons and the splice site consensus regions of *BTK*. The cohort included 117 patients with large B‐cell lymphoma (LBCL, this group included 46 patients with GCB DLBCL, 54 patients with non‐germinal center (non‐GC) DLBCL, five patients with non‐specified DLBCL, and 12 patients with high‐grade B‐cell lymphoma), 64 patients with follicular lymphoma (FL), 45 patients with LPL, 20 patients with MCL, 20 patients with marginal zone lymphoma (MZL), four patients with hairy cell leukemia (HCL), four patients with Burkitt's lymphoma (BL), 24 patients with unspecified non‐FL indolent B‐cell lymphoma, and 22 patients with T‐cell lymphoma (TCL). Patients with more than one sample analyzed were listed based on the first analyzed sample except patients with concomitant FL and LBCL samples, which were listed as LBCL. The patients were diagnosed by experts in hematopathology according to the WHO classification (revised 4th ed., 2017) [9]. In DLBCL, GCB and non‐GC subtypes were determined by immunohistochemistry based on CD10, BCL2, and MUM1 staining according to the Hans algorithm. *BCL2*, *BCL6*, and *MYC* translocation status was determined by fluorescence in situ hybridization (FISH). *BTK* mutations were classified using Varsome [10], literature review, and BTKbase [11]. To compare the characteristics of patients with and without *BTK* mutations, Fisher's exact test was used, and *p* < 0.05 was considered statistically significant. Data analysis was performed using R statistical software (Foundation for Statistical Computing, Vienna, Austria).

## 3 RESULTS AND DISCUSSION

Mutations in the *BTK* gene were found in 9/117 (7.7%) patients diagnosed with LBCL and in 9/64 (14.1%) patients diagnosed with FL (Table 1). *BTK* mutations were not detected in patients with LPL, MCL, MZL, HCL, BL, TCL, or unspecified non‐FL indolent B‐cell lymphoma. None of the patients with LBCL or FL was previously treated with BTK inhibitors. In LBCL, 8/9 (88.9%) of the patients with *BTK* mutations were of the GCB subtype according to the Hans classification, and no pathogenic or likely pathogenic *BTK* mutations were found among non‐GC patients, which substantiates the dependence of ABC LBCL on BTK signaling. The male to female ratio of the *BTK* mutation‐positive patients was 2 (12 male/6 female).

**TABLE 1:** Mutations in Bruton's tyrosine kinase (BTK)

Diagnosis	Number of patients	BCL2 translocation	Mutation^a^	Predicted protein	Classification	Comment
FL	6 male/3 female	5 positive/4 unknown	c.241‐2A>G	p.?	Likely pathogenic	No citation, not in BTKbase
			c.19G>C	p.Glu7Gln	Variant of unknown significance	No citation, not in BTKbase
			c.473_474delCA	p.Thr158fs	Likely pathogenic	No citation, not in BTKbase
			c.586C>T	p.Gln196Ter	Likely pathogenic	Previously reported in XLA^b^
			c.818A>T	p.Glu273Val	Variant of unknown significance	No citation, not in BTKbase
			c.1363G>T	p.Glu455Ter	Likely pathogenic	No citation, not in BTKbase
			c.1559G>A	p.Arg520Gln	Likely pathogenic	Previously reported in XLA^b^
			c.1574G>A	p.Arg525Gln	Likely pathogenic	Previously reported in XLA^b^
			c.1765_1766insG	p.Glu589fs	Likely pathogenic	No citation, not in BTKbase
LBCL	7 male/2 female	All positive	c.26T>A	p.Ile9Asn	Variant of unknown significance	No citation, not in BTKbase
			c.426T>A	p.Tyr142Ter	Likely pathogenic	Previously reported in XLA^b^
			c.599A>T	p.Lys200Met	Variant of unknown significance	No citation, not in BTKbase
			c.1030T>C	p.Tyr344His	Likely pathogenic	Previously reported in XLA^b^
			c.1573C>T	p.Arg525Ter	Likely pathogenic	No citation, not in BTKbase
			c.1578delC	p.Cys527fs	Likely pathogenic	Previously reported in FL^b^
			c.1753G>T	p.Val585Phe	Likely pathogenic	Previously reported in XLA^b^
			c.1881T>G	p.Tyr627Ter	Likely pathogenic	Previously reported in XLA^b^ and FL^b^
			c.1893C>G	p.Tyr631Ter	Likely pathogenic	Previously reported in XLA^b^

Abbreviations: FL, follicular lymphoma; LBCL, large B‐cell lymphoma; XLA, X‐linked hypogammaglobulinemia.

^a^ Reference sequence NM_000061.2.

^b^ References for previously reported mutations are listed in Table S1.

All *BTK*‐mutated LBCL patients showed *BCL2* translocation as determined by FISH. Among the LBCL patients without *BTK* mutations, *BCL2* FISH results were available for 105 patients. Only 20 of these patients harbored *BCL2* translocations. Thus, *BTK* mutation among LBCL patients was significantly correlated with *BCL2* translocation (Fisher's exact test, *p* < 0.00001). BCL2 translocation is found very frequently in patients with FL and often in transformed LBCL; however, the correlation between *BTK* mutation and *BCL2* translocation was also significant when only patients with de novo LBCL (non‐transformed LBCL, *n* = 4) were included (Fisher's exact test, *p* = 0.00191). BCL2 translocations were more frequent among DLBCL and HGL patients of the GCB subtype. Therefore, the correlation between BTK mutation and BCL2 translocation was also analyzed for the GCB subtype patients separately. With no BTK‐mutated patients without BCL2 translocation, eight BCL2 translocation‐positive/BTK‐mutated patients, 18 BCL2 translocation‐positive/BTK mutation‐negative patients, and 32 BCL2 translocation‐negative/BTK mutation‐negative patients, the correlation between BTK mutation and BCL2 translocation was still significant when only patients of the GCB subtype were included (Fisher's exact test, *p* = 0.000815). Altogether, our data suggest a strong coexistence of BTK mutations with BCL2 translocation; however, due to the small sample size of the BTK‐mutated subgroup, the association should be confirmed in another cohort.

The vast majority of *BTK*‐mutated FL and LBCL patients (78%) had mutations classified as likely pathogenic due to the introduction of termination codons, frame shift mutations, aberrant splicing or missense mutations classified as deleterious based on previous reports in patients with XLA (Table 1). Nonsense or frameshift mutations represented 53% of the detected variants. Only four *BTK* mutations were classified as variants of unknown significance. This selective bias for loss‐of‐function mutations could indicate that inactivation of BTK is an active mechanism in some B‐cell lymphomas rather than a passenger mutation phenomenon.

Inactivating mutations in *BTK* have previously been described in patients with FL and transformed DLBCL [12, 13]. Two of the mutations found in our LBCL cohort were also reported in patients with FL by Krysiak et al. [12], whereas the rest of the mutations found in our study were not previously described in FL or transformed LBCL patients (Table 1). Four (44.4%) of the *BTK‐*mutated LBCL patients in our cohort were not known to be transformed from FL, and inactivation of BTK therefore seems to occur de novo among LBCL patients as well, even though undiagnosed primary transformed FL cannot be completely excluded.

Hu et al. [13] recently presented a functional study of inactivating *BTK* mutations from FL patients and found that BCR activation in BTK inactivated or BTK deprived cell lines leads to increased AKT (protein kinase B) phosphorylation compared to cell lines with functional BTK. These results support that inactivation of BTK could be an active mechanism, even though the mechanism for increased AKT activation by BTK inactivation is still unknown. Interestingly, CXCR4 mutation, which is correlated with decreased sensitivity to BTK inhibition with ibrutinib in patients with LPL, also causes increased AKT activation and subsequent MAPK1/2 signaling [14]. Of note, secondary *BTK* mutations that confer resistance to BTK‐inhibitory therapy in CLL have recently been reported to support continuous AKT activation as well [15].

## 4 CONCLUSION

Inactivating mutations in *BTK* are present in a subset of LBCL patients naïve to BTK inhibitor treatment. Our data suggest a strong coexistence of these mutations with *BCL2* translocation. *BTK* mutations in FL and LBCL are distributed throughout the gene, and we therefore suggest that *BTK* sequencing in patients with FL and LBCL should include all exons and splice site consensus regions. In LBCL, inactivating BTK mutations were only found in GCB patients, which supports that the ABC subtype is dependent on BTK signaling. However, the absence of inactivating BTK mutations among ABC patients should be confirmed in larger patient cohorts.

## CONFLICT OF INTEREST

The authors declare they have no conflicts of interest.

## ETHICS STATEMENT

Data were collected retrospectively from patient files, and the study was approved as a Health Data Registry Study Research Project by the Capital Region of Denmark (journal no.: R‐ 21010067).

## FUNDING INFORMATION

The authors received no specific funding for this work.

## AUTHOR CONTRIBUTIONS

Lone Schejbel oversaw the molecular analyses. Marie Fredslund Breinholt and Peter Nørgaard performed pathological reviews. Anne Ortved Gang, Torsten Holm Nielsen, and Lars Møller Pedersen were in charge of patients. Lone Schejbel, Marie Fredslund Breinholt, and Anne Ortved Gang collected the data. Lone Schejbel wrote the paper, and Marie Fredslund Breinholt, Anne Ortved Gang, Torsten Holm Nielsen, Lars Møller Pedersen, Estrid Høgdall, and Peter Nørgaard revised it critically. All authors approved the final version of the manuscript.

## Supporting information

Supporting Information

## Data Availability

The data that support the findings of this study are available from the corresponding author upon reasonable request.
