# Supplementary material for: Inactivating BTK mutations in large B‐cell lymphoma in a real‐world cohort: Strong correlation with BCL2 translocation
Source: EJHaem. 2022 Jun 24;3(3):936–9. doi: 10.1002/jha2.489 (PMC9421985; doi:10.1002/jha2.489)
Supplement: Supplementary file 1 — Supporting Information [file JHA2-3-936-s001.pdf]

Supplementary Table SI. Citations for previously reported mutations

Mutations previously reported	Citation
p.Tyr142Ter	Gaspar, Lester, Levinsky & Kinnon. Bruton’s tyrosine kinase expression and activity in X-linked agammaglobulinaemia (XLA): the use of protein analysis as a diagnostic indicator of XLA. Clin. Exp. Immunol. 111, 334–338 (1998)
p.Gln196Ter	Rodríguez, M. C., Granados, E. L., Cerdán, A. F. & Casariego, G. F. Molecular analysis of Bruton’s tyrosine kinase gene in Spain. Hum. Mutat. 18, 84 (2001).
p.Tyr344His	Yeh, Y. H. et al. Distinct Clinical Features and Novel Mutations in Taiwanese Patients With X-Linked Agammaglobulinemia. Front. Immunol. 11, (2020).
p.Arg520Gln	Fiorini, M. *et al.* BTK: 22 Novel and 25 Recurrent Mutations in European Patients With X-linked Agammaglobulinemia. *Hum. Mutat.* Mar;23, 286 (2004).
p.Arg525Gln	>30 Citations, first published by Vetrie, D. et al. The gene involved in X-linked agammaglobulinaemia is a member of the src family of protein-tyrosine kinases. Nature 361, 226–233 (1993)
p.Cys527fs	Krysiak K, Gomez F, White BS, Matlock M, Miller CA, Trani L et al. Recurrent somatic mutations affecting B-cell receptor signaling pathway genes in follicular lymphoma. Blood 2017; 129: 473–483.
p.Val585Phe	Kanegane, H. et al. Clinical and mutational characteristics of X-linked agammaglobulinemia and its carrier identified by flow cytometric assessment combined with genetic analysis. J. Allergy Clin. Immunol. 108, 1012–1020 (2001).
p.Tyr627Ter	**XLA:** Fiorini, M. *et al.* BTK: 22 Novel and 25 Recurrent Mutations in European Patients With X-linked Agammaglobulinemia. *Hum. Mutat.* Mar;23, 286 (2004).<br>**FL:** Krysiak K, Gomez F, White BS, Matlock M, Miller CA, Trani L et al. Recurrent somatic mutations affecting B-cell receptor signaling pathway genes in follicular lymphoma. Blood 2017; 129: 473–483.
p.Tyr631Ter	Chen, X. F. et al. Clinical characteristics and genetic profiles of 174 patients with X-linked agammaglobulinemia: Report from Shanghai, China (2000-2015). Med. (United States) 95, (2016).
